# An initial machine learning model applied to local field potential data from the subthalamic nucleus to detect freezing of gait in Parkinson’s disease

**DOI:** 10.3389/fneur.2026.1793291

**Published:** 2026-05-19

**Authors:** Jay L. Alberts, Paul Cantlay, Anson B. Rosenfeldt, Christina Felix, Colin Waltz, Andrew S. Bazyk, Visar Berki, Nitesh Malan, Sean Nagel, Benjamin Walter, James Liao, David Escobar, Kenneth B. Baker, Mandy Miller Koop

**Affiliations:** 1 Department of Biomedical Engineering, Cleveland Clinic Research, Cleveland Clinic, Cleveland, OH, United States; 2 Center for Neurological Restoration, Neurological Institute, Cleveland Clinic, Cleveland, OH, United States; 3 Department of Neurosciences, Cleveland Clinic Research, Cleveland Clinic, Cleveland, OH, United States

**Keywords:** deep brain stimulation, freezing of gait, local field potentials, machine learning, Parkinson’s disease

## Abstract

**Introduction:**

Freezing of gait (FOG) is an unpredictable and debilitating symptom of Parkinson’s disease (PD). Current approaches to treating FOG are lacking; adaptive deep brain stimulation (aDBS) holds promise in treating FOG. However, a necessary precursor to using aDBS is understanding changes in the subthalamic nucleus (STN) prior to and during FOG. The aim of this project was to develop a machine learning model to detect FOG episodes using local field potential (LFP) data from the STN in individuals with PD.

**Methods:**

The LFP data were collected from five individuals with PD using the Medtronic Percept DBS platform while in the off-therapy state (off-DBS and off-medication) as they walked through virtual reality environments designed to induce FOG by manipulating levels of physical, anxiety, and cognitive load. The LFP time-series data were z-score normalized within each participant and canonical frequency band powers, rolling means, maxima, minima, and standard deviations were calculated. In addition, alpha-beta burst dynamics were calculated and included for analysis. The model was trained and subsequently tuned with a transfer learning component before testing its capacity to detect FOG episodes.

**Results:**

The deep learning model achieved an average weighted F1 score of 0.67, a macro F1 score of 0.62 across eight trials from five participants and successfully detected 24/29 (80%) of FOG episodes. The predictive importance of the twelve LFP channels varied across participants and FOG triggers, underscoring the need for an individualized, adaptable approach to modelling FOG using only LFP data.

**Discussion:**

Initial results indicate that FOG detection using LFP data gathered while walking is feasible. Future aDBS applications should contemplate a patient- and environmental-specific approach to optimize FOG treatment potential.

**Trial registration:**

clinicaltrials.gov, Identifier (NCT05103384).

## 1 Introduction

Gait impairment is a hallmark symptom of Parkinson’s disease (PD). As the disease progresses, freezing of gait (FOG) becomes more prevalent. Frequently described as “having one’s feet glued to the floor”, FOG is associated with falls (1) and results in decreased quality of life (2). Unfortunately, FOG is difficult to treat. Medication aimed to prevent FOG becomes less effective as PD advances, and a subset of patients exhibiting FOG are unresponsive to dopaminergic medication (3). Deep brain stimulation (DBS) has limited and mixed impact on treating FOG (4**–**6) with several reports noting a worsening of FOG following DBS (7**–**9). Contributing to the gap in treating FOG is a lack of understanding the precise role of the basal ganglia in FOG. Advancements in DBS technology (10) provide an opportunity to directly monitor and understand patterns of neural activity within the subthalamic nucleus (STN) which is necessary to realize the potential to use adaptive DBS (aDBS) to effectively treat FOG.

The safety and potential value of aDBS in treating PD tremor, bradykinesia and rigidity are well documented (11**–**13). A personalized aDBS approach aims to deliver stimulation through a closed loop system that modulates DBS stimulation in response to patient-specific pathological activity detected through local field potential (LFP) recordings (11) in contrast to conventional DBS systems that deliver continuous stimulation at a fixed electrical amplitude. Although both forms of stimulation are presumed to improve PD motor symptoms through the suppression of oscillations in the beta band (13–30 Hz), aDBS has been shown to selectively target prolonged beta bursts (14) which has been correlated with greater PD disease severity, bradykinesia and rigidity (15, 16). In contrast to conventional DBS which broadly suppresses beta band activity, aDBS preserves short-duration beta bursts (14), now considered integral to normal motor function (17) and associated with improvements in overall disease state (15). These findings are supported by a recent systematic review and meta-analysis that concluded aDBS improved overall PD motor symptoms, specifically rigidity, bradykinesia, dyskinesia, and speech function comparably to conventional DBS, while using significantly less electrical stimulation (18). There was insufficient data to draw conclusions regarding aDBS and its effectiveness in treating gait dysfunction (18).

Preliminary reports suggest that the basal ganglia play a dynamic, modular role throughout the gait cycle (19) and during FOG (20–22). At rest, differences between beta activity in people with PD with and without FOG is mixed with some reporting an increase in beta activity in freezers compared to non-freezers (21, 23) and others reporting no differences in beta power or entropy between those with and without FOG (24). During movement, LFP signals appear to differ in freezers and non-freezers. During both cycling and walking, non-freezers displayed a power reduction throughout the beta band; freezers experienced a similar power reduction in the overall beta band; however, a slight power increase in a narrow band around 18 Hz was present with movement initiation (21). Singh reported a reduction of beta oscillations in non-freezers during walking compared to standing or sitting. By contrast, freezers had an increase of low beta oscillations during walking (20). During a stepping in place task, Syrkin**–**Nikoula reported lower beta power, but greater beta entropy in the STN in freezers compared to non-freezers (24). Others report enhanced STN beta and theta band synchronization in people with PD during FOG (25) and greater low beta and theta power LFP power during periods of vulnerable gait, or where the FOG index is high and FOG is likely to occur (26), suggesting that there may be a neural signal associated with FOG episodes. The preliminary results characterizing the neural signature underlying FOG provide rationale for monitoring and recording LFP data across a range of environmental stimuli to enhance the potential treatment of FOG using aDBS. The aim of this project was to determine if STN LFP data gathered during walking through a series of virtual environments designed to trigger FOG could be used to inform a machine learning model that could aid in the detection of FOG. Identifying an effective machine learning model approach using only STN LFP data has the potential to provide the cornerstone for the development of an effective aDBS approach to treating FOG.

## 2 Materials and methods

Individuals with PD who previously (>3 months) underwent bilateral STN DBS surgery (Percept™ DBS, Medtronic, Inc.) were eligible for participation. Additional inclusion criteria were: the ability to ambulate with or without an assistive device for at least 10 min and a score of ≥1 on the Freezing of Gait Questionnaire (27). Exclusion criteria were documented clinical diagnosis of dementia, neurological disease other than PD including multiple sclerosis, stroke, and epilepsy, musculoskeletal issues that would prevent prolonged standing or ambulation, untreated mood disorder, active substance abuse, and uncorrected hearing or vision impairment. Participants completed the informed consent process approved by the Cleveland Clinic Institutional Review Board prior to data collection.

**Figure 1:**
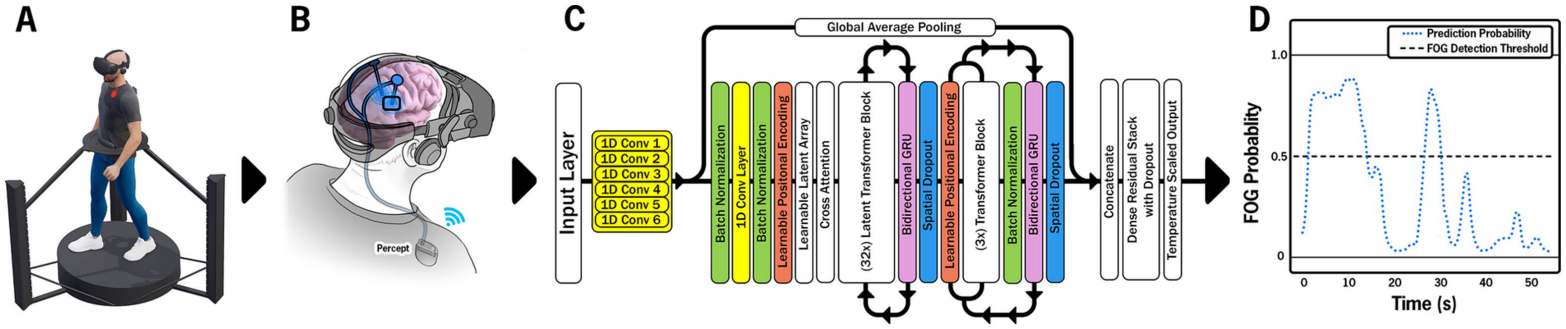
**(A)** Participant with PD and DBS walked through the Cleveland Clinic-Virtual Home Environment (CC-VHE) in the OFF-therapy state. The CC-VHE consists of three modules with varying environmental stimuli intended to induce FOG: physical changes in the environment, anxiety provoking and cognitive loading. **(B)** LFP data were recorded and streamed from the electrode within the STN. **(C)** The LFP data were input into the deep learning model. The model employs a transfer-learning approach to customize model weights for each CC-VHE trial. A series of machine learning layers were used to optimize temporal reasoning for accurate FOG detection. **(D)** The FOG probability estimate on an individual trial basis was identified by the model following training and transfer-learning.

Data were gathered from five individuals with PD in the OFF-therapy condition defined as withholding antiparkinson medication >12 h and DBS off for ~1 h. Participants completed the Cleveland Clinic-Virtual Home Environment (CC-VHE) while LFP data were streamed and recorded from the electrodes in the STN. An overview of the data collection and processing is provided in Figure 1. Participant demographics are provided in Table 1.

**Table 1:** Participant demographics.

	Age, years	Race	Ethnicity	Gender	Years of education	Years since PD diagnosis	Years since DBS surgery	MOCA score (out of 30)	FOG-Q Score	Hoehn & Yahr OFF-med/OFF-DBS	MDS-UPDRS III OFF-med/ OFF-DBS	Levodopa equivalent daily dose (mg)
Subject 1	65	White	Not Hispanic or Latino		Male	18	6	0.75	22	19	3	59	1,150
Subject 2	62	White	Not Hispanic or Latino		Male	14	5.5	0.5	27	6	3	75	275
Subject 3	71	White	Not Hispanic or Latino		Male	16	20	0.5	27	8	2	38	825
Subject 4	63	White	Not Hispanic or Latino		Female	14	5	0.5	28	11	3	30	832.5
Subject 5	68	Two or more races	Hispanic or Latino		Male	20	8	0.5	27	8	3	64	200

FOG-Q, freezing of gait questionnaire; MDS-UPDRS, movement disorder society—unified Parkinson’s disease rating scale; MoCA, Montreal cognitive assessment; PD, Parkinson’s disease.

### 2.1 Cleveland clinic – virtual home environment (CC-VHE) paradigm

The CC-VHE platform combines an omni-directional treadmill with immersive virtual reality scenarios to create real-world scenarios known to provoke FOG. Each participant completed three CC-VHE modules, previously detailed (28). The modules were developed to operationalize theoretical models of FOG: (1) Physical (29), (2) Anxiety (30), and (3) Cognitive (31). Briefly, the Physical module changes the external characteristics of the environment including different flooring patterns, narrow doorways and hallways, and multiple turns. The Anxiety module aims to evoke a stress response by requiring the participant to walk on a narrow plank suspended high above the ground. Finally, the Cognitive module requires dual-tasking as the participant ambulates around a large kitchen island while responding to a picture interference task (i.e., identify whether the picture of the animal is congruent or incongruent with the text). The Physical and Anxiety modules required walking ~30 meters while the Cognitive module had a trial time of 2 min of continuous walking. The participants completed one trial of the Physical, Anxiety, and Cognitive modules.

### 2.2 Local field potential recording

Bilateral STN LFP data were continuously recorded at 250 Hz using Indefinite Streaming Mode. Data from three channel pairs, LFP_0–2_, LFP_0–3_, LFP_1–3_ for each STN, were streamed wirelessly and stored on the programmer tablet (Model CT900D, Medtronic). The data were passed through built-in filters prior to streaming including a low pass filter with a cutoff frequency of 100 Hz, and a high pass filter with a cutoff frequency of 1 Hz (32). The filtered LFP magnitude (μV) in the time domain were downloaded as a JSON file for offline analysis in MATLAB version R2022b (MathWorks, Inc., Natick) and Python version 3.11.3.

### 2.3 Data synchronization and freezing of gait detection

Our previously described methods of data synchronization ensured LFP and gait data were synchronized to within 50 ms (28). The methodology leveraged an electroencephalogram (EEG) system to synchronize the CC-VHE (i.e., FOG events) and LFP data. While EEG data were collected as part of a larger project, these data are not presented in this study as the focus of this manuscript is detecting FOG using only the LFP data. Briefly, the STN LFP and EEG data were synchronized using a commercially available TENS device (BioStim Plus; Biomedical Life Systems), which injected an electrical artifact into both data streams prior to the start of the module. A physical therapist (Board-Certified Clinical Specialist in Neurologic Physical Therapy with 15 + year of clinical and research experience with PD) operated the control computer to (1) initiate and terminate each trial and (2) to mark the start and stop times of each FOG episode in real-time (termed clinician-identified FOG events). The EEG system (LiveAmp 64; Brain Vision) recorded the two aforementioned events as well via a hardwired connection with the control computer. The FOG start and end timepoints recorded in the EEG files were confirmed post-hoc via video review by the same physical therapist. Specifically, the onset of FOG was defined as akinesia or trembling resulting in non-functional steps (33); the offset of FOG was defined as two functional steps. In addition, the timeseries data from shank IMUs were visually inspected to verify clinically identified FOG episodes. The timepoints of the FOG episodes were identified in the LFP data following temporal synchronization of the EEG and LFP data streams and utilized for subsequent analysis.

### 2.4 Data feature engineering

Prior to modeling, features were derived and combined with individual participant LFP data. These data were then segmented to create input windows for model training and evaluation. All derived features were computed at each timepoint with preceding data, prior to segmentation into overlapping 3 second windows for input to the model.

#### 2.4.1 Additional channels and Z-score normalization

Raw LFP data from the 6 channels from the DBS leads were linearly combined to calculate 6 additional channels, 3 per side: LFP_0–1_ = LFP_0–3_ − LFP_1–3_; LFP_1–2_ = LFP_1–3_ + LFP_0–2_ − LFP_0–3_; LFP_2–3_ = LFP_0–3_ − LFP_0–2_. The additional channels were included in the analysis as they provided greater spatial resolution and enhanced sensitivity to localized neural changes during FOG events compared with exported channels, which spanned a larger anatomical area. Z-score normalization was performed on all 12 channels of time-series LFP data for each participant across their three trials. Accurate lead placement in the sensorimotor region of the STN was inferred using improvements in MDS-UPDRS III scores; reported improvement of ~30%–50% have been reported following DBS (34, 35). The mean standard deviation (SD) MDS-UPDRS III score for participants in this study in the OFF-DBS/OFF-Med state was 53.2 (18.6) compared to 33.2 (17.8) points in the ON-DBS/ON-Med state, representing an average improvement of 38%.

#### 2.4.2 Canonical frequency band powers

Power spectral densities were calculated using the Welch method (36) for the delta (1.0–4 Hz), theta (4–8 Hz), alpha (8–13 Hz), beta (13–30 Hz), and gamma (30–100 Hz) bands using 500, 250, 125, 62, and 31 samples, respectively. All band-power estimates were derived from a sliding window consisting of data preceding each timepoint, with the window advancing one timepoint per step.

#### 2.4.3 Custom frequency band powers

Power spectral densities (PSD) were calculated from at least 20 seconds of data collected while the participant was seated at rest prior to initiating CC-VHE modules and used to calculate participant-specific frequency bands. Specifically, PSDs were calculated using the Welch method with a 1 second Hanning window and 0.5 second overlap (36). A generalized power law (1/f^alpha^) function was applied to each channel’s PSD to simulate the physiological baseline activity in the brain. Peak power values, maximum value above the 1/f baseline in the alpha-beta band (8–30 Hz), were selected from the PSDs. The frequency corresponding to the largest peak power across all 6 channels was identified per participant per hemisphere. Custom frequency bands of 6, 10, and 20 Hz bandwidth centered at the frequency of the peak power per hemisphere were then determined and used to calculate power with the same methods described above used for canonical band power calculation for all channels per trial.

#### 2.4.4 Rolling features calculation

Rolling means, minima, maxima, and standard deviations were computed per timepoint on all features described in sections 2.4.1–2.4.3 using 0.125, 0.5, and 2 second sliding windows to highlight trends in LFP data leading up to and during changes in FOG state.

#### 2.4.5 Alpha-beta burst and duration

Participant-specific, resting-state alpha-beta burst thresholds were determined using methods described by Neuville et al. (16) which provides a broader range of burst durations than approaches based on high power thresholds (17). Notably, burst activity was not restricted to the beta band; alpha-beta frequencies (8–30 Hz) were included, consistent with emerging evidence linking both alpha and beta burst activity to FOG (37). To calculate burst durations, the resting-state LFP was bandpass filtered to produce five datasets per trial; each corresponding to a consecutive 6 Hz bandwidth spanning 45–63 Hz. The filtered LFP data were squared. Local minima were then extracted via interpolation, and the median value was computed per dataset. For each participant and channel, the average of the five median values was multiplied by 4 and used as a baseline threshold to calculate alpha-beta burst durations in the LFP data acquired during the modules. Specifically, for each module, the peak power in the alpha-beta band (8–30 Hz) and corresponding peak frequency in the PSD was identified for each participant, hemisphere, and channel. The data were then bandpass filtered using a 6 Hz bandwidth centered on the peak frequency and then squared. The amplitude envelope was estimated using the local maxima. Bursts were identified when the envelope of the signal exceeded the resting-state baseline threshold. Average burst duration was calculated on a per module, per participant, per hemisphere, per channel basis.

The features described in sections 2.4.1–2.4.5 totaled 1,020 features, split evenly between the 12 LFP channels. For pairs of highly correlated features (*R* > 0.95), one feature of each pair was dropped at random, with 970 features remaining for modelling. Data were then processed into model input windows for training and evaluation of FOG state.

### 2.5 Data processing

Data were processed into model input windows to support a deep learning model that predicts a timepoint’s binary state of FOG or non-FOG based on a short history of data immediately preceding it. *Training* the model involved passing in segmented windows of LFP data labeled with the binary outcome variable of FOG state of the final timepoint. The model also employs transfer learning (38), which is additional training, or *tuning*, of an existing model using labeled data closely related to the set to be predicted. Tuning assists an initial (or global) model that has already learned general features to refine its predictions in a specific (or local) setting. It is often used when there is a limited amount of closely related labeled data available for the target set.

**Figure 2:**
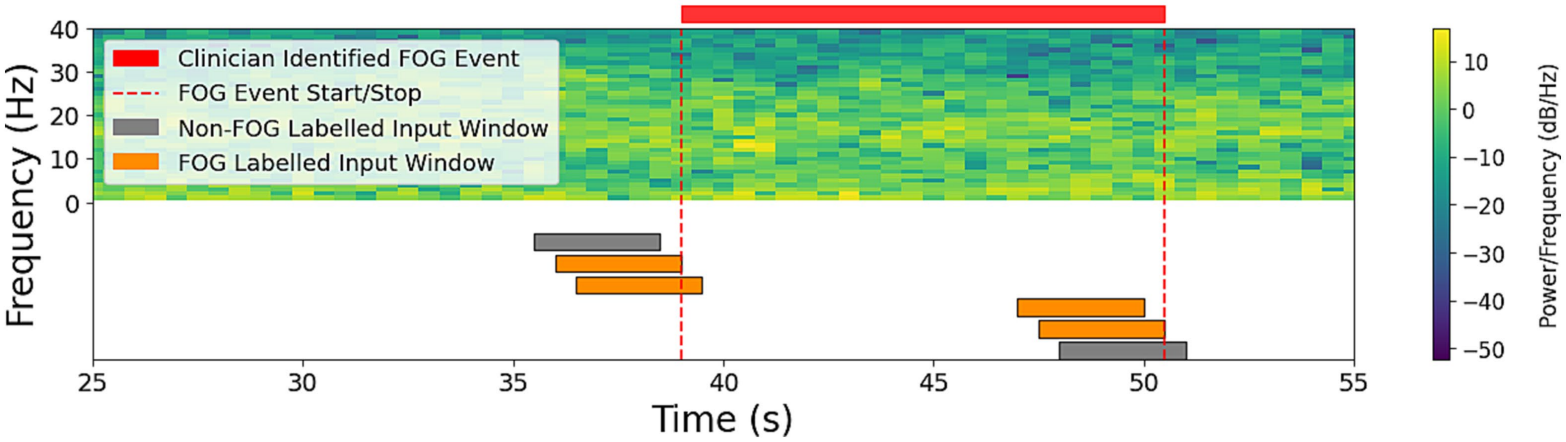
Clinician-identified FOG event, (red bar above the spectrogram) and corresponding input window labels (below the spectrogram). LFP data from a single participant are displayed in the spectrogram from the DBS channel with the greatest alpha-beta peak power. Data were processed into overlapping 3 second windows (orange and gray bars below spectrogram), each labeled according to the clinician-identified freeze state (red bar above spectrogram) at its final time point.

Each timepoint was labeled FOG or non-FOG according to the confirmed FOG start and stop times. The time series was segmented into 3 second sliding windows, with 0.5 second steps as shown in Figure 2. Downstream predictions were made by classifying the final timepoint of each 3 second window as FOG or non-FOG to evaluate performance. Predictions were made independently of subsequent data, reflective of a real-world environment where future data is unknown.

Data from each trial were truncated to include no more than 10 seconds of non-freeze prior to the first freeze and no more than 10 seconds of non-freeze following the final freeze for each trial to mitigate class imbalance. Class weights were also applied during model training to further address class imbalance.

LFP data were modeled and evaluated using leave one trial out cross validation (LOTOCV). For each LOTOCV iteration, one trial was held out from the data used to train an initial model. The held-out trial was partitioned into mutually exclusive tuning and testing sets; the first 40 seconds served as the tuning set while the remaining portion became the testing set used for performance evaluation. Held-out trials that did not result in at least one freeze in both their tuning and testing partitions after all processing and truncation were completed were excluded from modelling. The training set then consisted of (1) all remaining trials from the same participant as the held-out trial plus (2) all other participants’ trials from the same module as the held-out trial. The exclusion criteria ensured meaningful contributions from the trials included in the training and tuning sets, and interpretable results in the performance metrics from the held-out trials.

Tuning data were resampled with added noise to increase data set size prior to model tuning. Specifically, a Gaussian jitter with a standard deviation equal to 1% of the SD of each numeric feature was applied. Two augmented copies of the dataset were generated and concatenated with the original tuning data, ensuring sufficient variability for tuning without distorting the underlying time-series dynamics.

The test sets for the LOTOCV ranged from 33.5 to 77 seconds in length (Table 2), reflecting differences in overall trial duration and the amount of truncation necessary. In each LOTOCV iteration, these segments were evaluated for model performance using F1 score (an accepted measure of class-imbalanced model accuracy; range 0–1; higher values represent greater accuracy) to mitigate the impact of any remaining class imbalance on performance metrics.

**Table 2:** Results of model performance per segment and class breakdown of the analyzed trials.

ID	Module	Test Set Duration (seconds)	Number of Freezes	Freeze Duration (seconds)	% Test Trial Frozen	Weighted F1 Score	Macro F1 Score	Sensitivity	Specificity
Subject 1	Cognitive	51.5	3	33.5	65%	0.735	0.729	0.627	0.919
Subject 2	Cognitive	77	6	33.9	44%	0.511	0.510	0.536	0.488
Subject 2	Physical	33.5	5	18.2	54%	0.434	0.429	0.556	0.313
Subject 3	Cognitive	58	3	17.4	30%	0.638	0.609	0.694	0.593
Subject 4	Cognitive	59.5	1	13.5	23%	0.736	0.614	0.370	0.849
Subject 4	Physical	41.5	4	8.2	20%	0.761	0.649	0.529	0.806
Subject 5	Cognitive	65.5	6	29.3	45%	0.712	0.701	0.667	0.750
Subject 5	Anxiety	60.5	2	15.2	25%	0.797	0.744	0.767	0.793
Average/Sum		447	30	169.2	38%	0.666	0.624	0.593	0.689

*A total of five participants completed 15 modules. Based on model inclusion criteria requiring at least one freeze present in both the tuning and testing sets per module, eight trials across five participants were evaluated for model performance.

### 2.6 Model design

A deep learning model was designed to predict the final timepoint as FOG or non-FOG using the preceding 3 second window of time series LFP data and derived variables as input. Python packages Keras (version 3.6.0) and TensorFlow (version 2.16.2) were used. The model leverages a combination of several types of machine learning layers. The model architecture is designed using layers capable of capturing temporal dependencies and handling high-dimensional data, making it well-suited to learn meaningful patterns of FOG and non-FOG periods in neural data. A convolutional stack was chosen to learn local features across multiple time scales (39, 40). Positional encoding blocks assist the model to maintain sequence order and preserve temporal dependencies within the data. The cross-attention block was used because of its ability to focus on the portions of input sequences most relevant to prediction (41) and the ability to interpret temporal flow and understand global patterns in the data (42). Bidirectional Gated Recurrent Unit (GRU) layers model time-series data (43) by allowing the flow of information in both forward and backward directions. Transformer blocks enhance the model’s long-range temporal reasoning (44). Shortcut connections of the convolutional stack connect local and broader global signals together. Finally, a temperature-scaled output enhanced the confidence of model predictions. Transfer learning was implemented to assist the model in learning the heterogenous neural signatures of FOG across unique individuals and unique FOG triggers. This technique improved performance by splitting model training into two parts: the first, where it learns general, high level trends during training on data from the entire cohort, and the second, where data from the beginning of the held-out trial to be predicted is input to fine-tune the model weights and optimize prediction capability for the specific test set.

Collectively, this modelling architecture implemented with transfer learning enabled the model to capture low- and high-level features required to successfully predict FOG using only LFP data across several individuals and FOG triggers.

### 2.7 Model training

Data were trained for 100 epochs with a learning rate of 1×10^−3^ using an Adam optimizer and a binary focal cross entropy loss function with learning rate reduction on plateau and early stopping callbacks monitored by training loss and validation loss, respectively. Transfer learning was applied by freezing the first two layers of the model, reducing the learning rate to 5×10^−6^, and training the model for 100 additional epochs with data only from the held-out trial to fine-tune the model weights to the individual and FOG trigger in each iteration’s testing set. The same optimizer, loss function, and callbacks were used with the reduced learning rate during transfer learning.

### 2.8 Feature importance

Deep learning models process large numbers of individual inputs in a complex, non-linear manner, making calculation and interpretation of individual feature importances challenging. In this data, one 3 second input window contains 970 input features at 750 timepoints, for a total of 727,500 inputs per window, which pass through several layers of additional feature extraction, pattern recognition, and regularization, before the model outputs a prediction of the probability that the window ends in a FOG event. A feature importance approach was selected that leverages estimates of Shapley values (45) to quantify the marginal contribution of each input to the model’s prediction, then sums the values from related inputs into feature groups that are more easily interpretable.

Shapley values were estimated using the SHAP package (46) which approximates the values using a gradient-based approach and a set of background reference samples (46, 47), which were pulled from the training set. For a given input window and output prediction, the sum of the 727,500 individual contributions, or local SHAP values, combined with the model’s expected baseline output, equals the final prediction for that window. SHAP values were grouped across all inputs from the same channel and plotted against raw feature values to provide a high-level view of how the model predictions relate to input values.

## 3 Results

Five participants completed three modules each for a total of 15 completed trials. Of those 15 completed trials, 8 contained sufficient FOG to satisfy the requirements of at least one FOG each in the tuning and testing set and were therefore held out for one iteration of LOTOCV as the test set.

In total, the testing sets contained data from 30 FOG episodes (169 seconds) across all trials (Table 2). The model successfully detected 24 (80%) of the 30 freezes across the cohort with a specificity of 55%. A 0.5 prediction probability threshold was used to classify predictions as FOG or non-FOG. Successful detection of a FOG at the event level was defined as at least 25% of the predictions made per true FOG event at or above the chosen prediction probability threshold of 0.5. A 25% detection threshold was selected using ROC analysis by maximizing the Youden index (sensitivity + specificity − 1), subject to a minimum specificity constraint of ≥ 0.5 to ensure adequate classification of non-FOG events (48). This constraint was imposed to reflect the unequal costs of misclassification, with the minimum specificity determined by a clinician. The number of freezes containing at least one positive prediction within the duration of a FOG event was 26 (87%) of the 30 total freezes.

**Figure 3:**
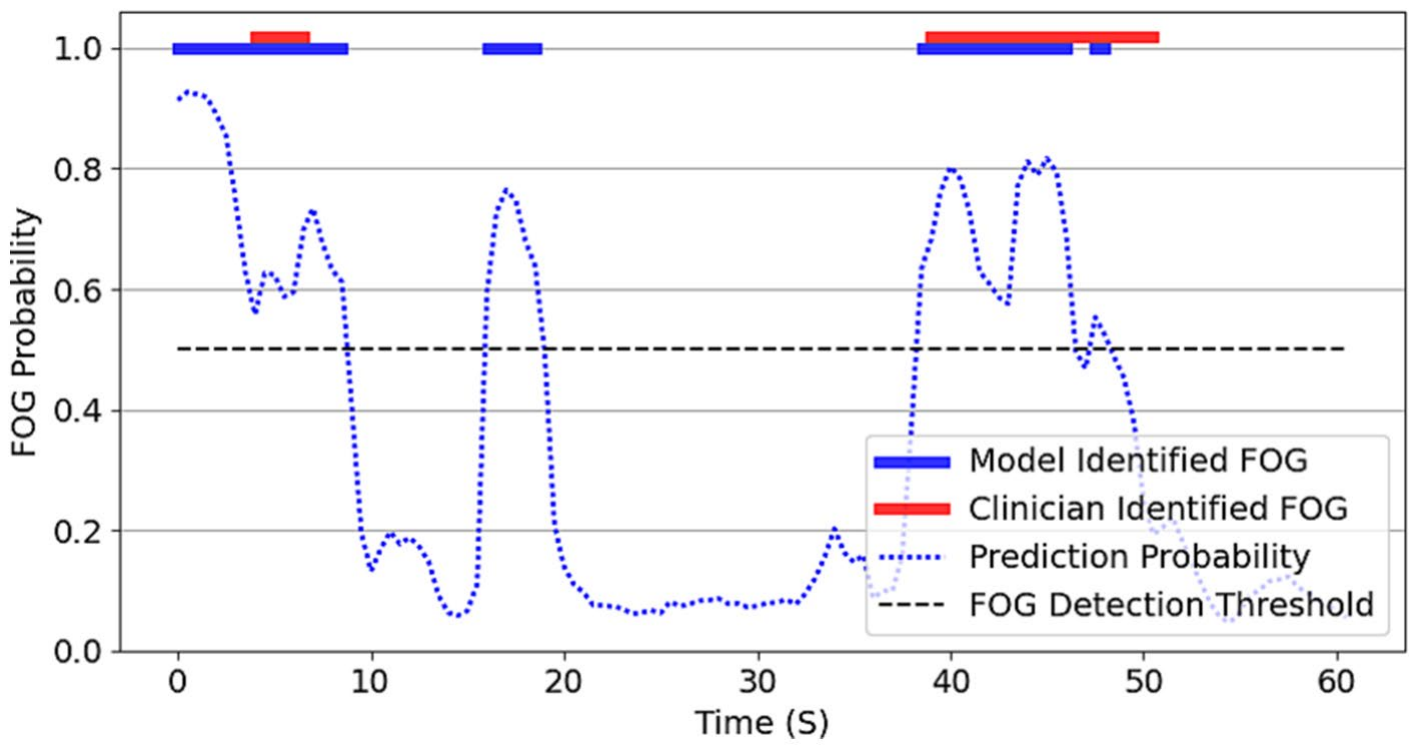
Model identified FOG vs. clinician-identified FOG in the anxiety module of one participant. The blue dotted line depicts model-predicted probability of a FOG episode, with probabilities of 0.5 + classified as a Model identified FOG (blue bar). Clinician-identified FOG is depicted by the red bars. Both the clinician and the model identified FOG episodes with high agreement. The smooth probability changes before FOG suggests subtle neural signature changes prior to clinical identification. Similarly, the model captured the gradual change in STN activity as the participant was coming out of the FOG.

Additional performance metrics were calculated per 0.5 second interval timepoint prediction using strict alignment between model identified and clinician-identified FOG. An example of a model-identified FOG compared to the clinician-identified FOG in a test set is shown in Figure 3.

In the testing sets, there were a total of 902, 3-second windows input into the transfer learning models for FOG prediction across iterations of LOTOCV; 38% of those windows ended in a FOG based on the clinician label of the final timepoint. Prior to applying transfer learning, cross-validated performance evaluation of the base model yielded an average weighted F1 score of 0.57 and an average macro F1 score of 0.51 on the test sets. After transfer learning, these scores improved to 0.67 and 0.62, respectively; a per participant breakdown and additional performance metrics of the transfer learning model are displayed in Table 2.

Across the 8 trials, the average precision was 0.55, and the average recall/sensitivity was 0.59. There was an average specificity of 0.69 across the trials. Of 166 total false positive predictions, 41 (25%) occurred within 2 seconds of the beginning of a clinician identified FOG with continuous positive prediction through the onset of the ensuing freeze – indicating early, rather than incorrect FOG detection. An additional 41 (25%) of the 166 false positives were continuous extensions of true positive predictions within 2 seconds of the end of a clinician identified FOG. In summary, approximately half of all false positives occurred within 2 seconds of clinician-identified FOG events, appearing to reflect temporal overextension surrounding true positive detections.

**Figure 4:**
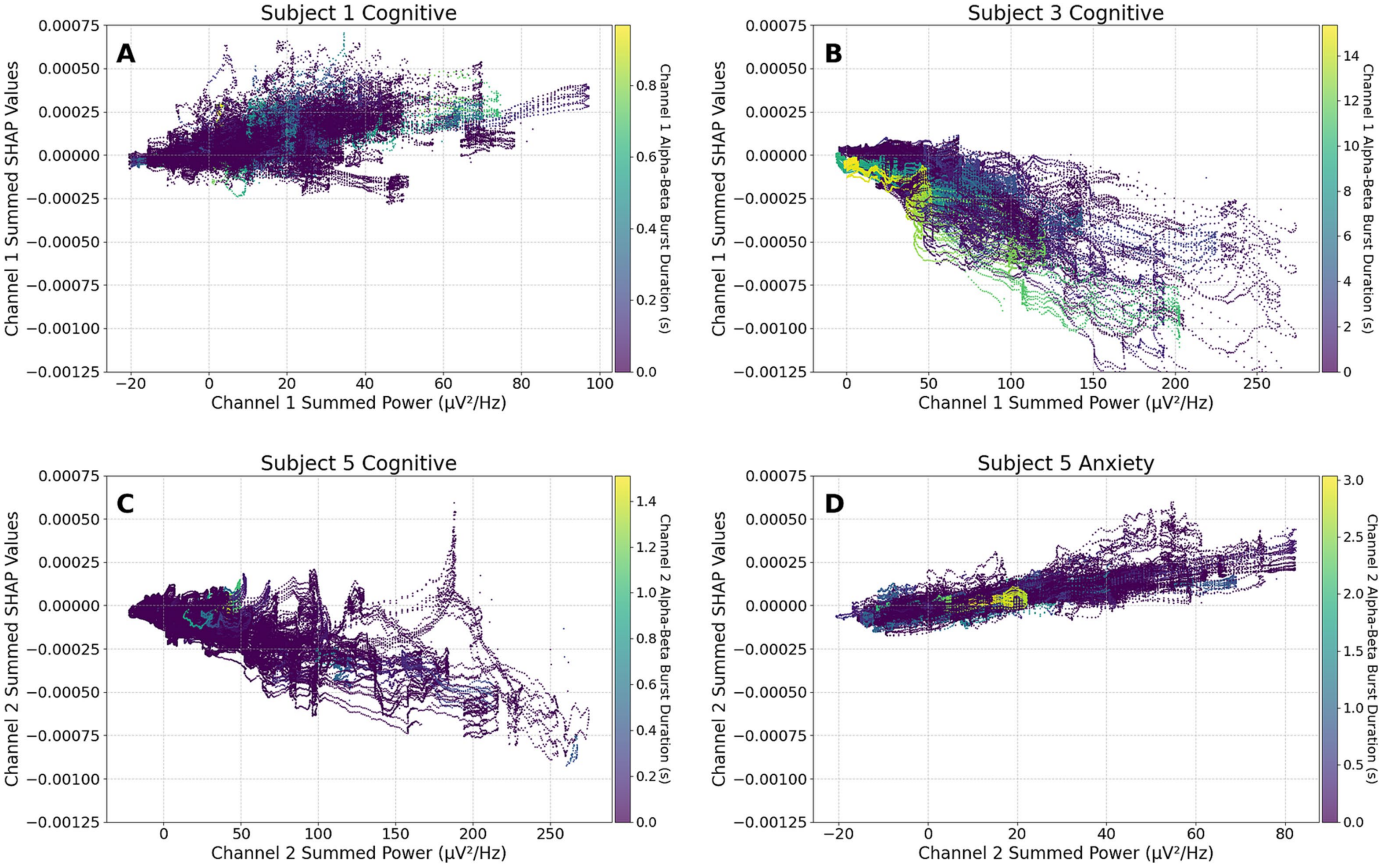
SHAP partial-dependence summaries. Each graph plots the sum of raw feature values on the x-axis against the sum of the corresponding SHAP values on the y-axis for a single LFP channel (LFP0_2_Left for A–B; LFP2_3_Right for C–D) for each timepoint (750 per window; between 105 and 132 windows). The feature subset summed is comprised of canonical band powers (delta, theta, alpha, beta, gamma) plus the custom 6, 10 and 20 Hz bandwidth power bands described above, and their respective rolling metrics. Point color shows the channel’s alpha-beta burst duration (channel-specific color bar at right of each panel). Panels **(A)** and **(B)** contain the sum of the raw feature values for Subjects 1 and 3 during the Cognitive module. Despite the two participants performing the same module, feature importance varied significantly, highlighting between-subject heterogeneity. Panels **(C)** and **(D)** are from a single subject performing two different modules, Cognitive and Anxiety. The differences in feature importance highlight the heterogeneity within a single subject presented with varying stimuli.

The variability in neural signatures across individuals and FOG triggers, preceding and during freezing, is shown in Figure 4. Each subplot shows the sum of the canonical band powers (delta, theta, alpha, beta, gamma) plus the custom 6, 10, and 20 Hz bandwidth power bands, and their respective rolling metrics on the x-axis plotted against the sum of their corresponding SHAP values on the y-axis for a single LFP channel.

In Figures 4A,B, the raw values and feature importance values from the cognitive trials of two different subjects are plotted adjacent to one another. Feature impact between the two models has an inverse relationship. For subject 1 in Figure 4A, increased feature values lead to a higher predicted probability of a FOG event. For subject 3 in Figure 4B, however, increased feature values led to a decrease in the predicted probability of a FOG event. The inverse relationship between Figures 4A,B illustrates the between-subject variability in neural signatures across individuals.

In the second row, displaying Figures 4C,D, a similar pattern is seen between two different FOG triggers within the same individual. Both models’ feature importances are plotted against raw feature values for one channel of the same subject with different FOG triggers. In Figure 4C, subject 5’s cognitive trial, raw feature values and SHAP values show a negative correlation, meaning increased feature values result in lower predicted FOG probability. Conversely, in Figure 4D, subject 5’s Anxiety trial, a positive correlation is observed between feature importance and raw values, signaling an increased likelihood of model predicted FOG as feature values rise. The difference in neural signature is unique not only between individuals, but also across FOG triggers within individuals. Together, these patterns highlight the subject- and trigger-specific nature of neural signatures associated with freezing, highlighting the importance of a customized, fine-tuned approach to reliably predict FOG events with LFP data alone.

## 4 Discussion

Results from this project support the feasibility of using LFP data to detect FOG events while walking through VR environments that replicate those in the real-world known to trigger FOG. Several findings from this study inform future LFP FOG detection machine learning algorithms. First, the environmental stimuli or FOG trigger appears to be an important factor in model accuracy. Unique environmental triggers (i.e., physical, anxiety, and cognitive) produced differing feature importance magnitude and directionality of channel feature values, suggesting a unique pattern of neural data between environmental triggers. Second, FOG-related changes in neural activity appear to vary across individuals. To address the importance of the environmental stimuli and individual signal, we developed individual models using custom metrics and transfer learning. Custom frequency bands were defined to capture each participant’s peak power rather than relying on uniform canonical bands, as diverse neural signatures limit the comparability of standard power metrics. The patient-specific bands enable the model to directly compare peak power across individuals. Leveraging comprehensive LFP frequency bands, not just beta, is consistent with previous findings related to the development of a neural network model to predict weight shifting during a rhythmic stepping in place activity in PD (49). This data set provides an initial signal that FOG events produce a highly individualized, stimuli-specific neural signature.

Transfer learning was used to personalize predictions and increase performance beyond the base models trained on data from multiple participants and FOG triggers. The LFP data from five participants was used to create individual base models. Although each base model captured mild signal, they lacked the precision necessary for accurate, individual predictions. The base models were tuned on a single trial’s dataset to create personalized, trigger-specific LFP FOG detection. Marked improvement in predictive performance across all participants and all FOG triggers was present after fine-tuning model weights during transfer learning (0.57 vs. 0.67 weighted F1 score), underscoring the diversity of neural signatures across participants and triggers and highlighting the need for an individualized modelling approach in the absence of lower extremity kinematic and accelerometer data. The transfer learning approach is a feasible model for clinical care, as a general model trained on a large dataset of FOG events could be used as a base model for all patients, with transfer learning to the individual occurring at the same time as the initial DBS programming session.

Currently, the gold-standard of identifying FOG is through clinician assessment of gait behavior; this approach may be prone to error. Individual FOG patterns can present as akinetic, trembling (knees or lower shank), and festinating (small, non-functional steps) (33, 50), which makes visual FOG detection challenging and subjective. Despite recent work to standardize the definition and features of FOG (33), variability remains due to the nature and temporal occurrence of FOG presentations which can compromise intra- and inter-rater consistency. In the present study, model performance was evaluated using FOG episodes in which alignment between clinician-identified and model-identified FOG were present, resulting in conservative model performance estimates. For example, in Figure 3, the model-identified FOG preceding both freezes are scored as false positives, despite representing clinically advantageous early detection. Similarly, false positives following the first freeze and false negatives following the second freeze in Figure 3 weaken performance metrics regardless of their minimal practical impact in a real-world environment. Greater standardization through the consensus definition and utilizing biomechanical data to objectively identifying FOG will improve accuracy and reliability of subsequent FOG detection models.

This project included extensive walking and freezing data in multiple realistic virtual environments from a relatively small number of participants, resulting in multiple personalized initial and tuned models. The protocol validated that the CC-VHE platform is an effective tool for collecting FOG data in a clinical setting, reliably inducing FOG from multiple triggers. The success in data collection and personalized modeling supports the pursuit of a broader data collection effort. With additional subjects, a single base model could be trained on a comprehensive dataset across many individuals and FOG triggers, eliminating the need for individualized base models. Further exploration of neural signatures associated with FOG triggers within a specific module (i.e., turning vs. narrow hallway in the Physical module) will be possible with a larger dataset. Refinement of the machine learning model, including adding aperiodic components (49), additional high-quality data, and the implementation of a single generalized base model will likely improve and refine performance.

Current on-demand FOG detection models predominately utilize external sensors integrated with other technologies to deliver an intervention, such as auditory, visual, or sensory cues (51). On-demand cuing technologies may be effective in reducing FOG (51); however, there appears to be no carryover when the device is removed (52) and implementation barriers such as external device wear compliance, comfort, appearance, technology failure, and charging requirements limit adoption. Adaptive DBS can alter stimulation in response to a detected electrical stimuli, thus the potential for disrupting a pathological FOG signal and preventing FOG. Our preliminary data demonstrate that FOG detection is feasible and that consideration of individual LFP data and the FOG trigger is paramount. While preliminary, the modelling approach provides rationale for moving forward with continued investigation into aDBS to detect and potentially treat FOG.

## Data Availability

The raw data supporting the conclusions of this article will be made available by the authors, without undue reservation.
